# 肺混合密度磨玻璃结节中临床影像学信息对淋巴结转移的预测价值

**DOI:** 10.3779/j.issn.1009-3419.2023.101.06

**Published:** 2023-02-20

**Authors:** Jian GAO, Qingyi QI, Hao Li, Jie YU, Jian ZHANG, Bingbing LIN, Xiao LI, Nan HONG, Yun LI

**Affiliations:** ^1^100044 北京，北京大学人民医院胸外科，北京大学人民医院胸部肿瘤研究所（高健，李浩，张建，李晓，李运）; ^1^Department of Thoracic Surgery, Thoracic Oncology Institute; ^2^放射科（齐清怡，林冰冰，洪楠）; ^2^Department of Radiology, Peking University People’s Hospital, Beijing 100044, China; ^3^266034 青岛，青岛市妇女儿童医院（于洁）; ^3^Qingdao Women and Children’s Hospital, Qingdao 266034, China

**Keywords:** 混合密度磨玻璃结节, 淋巴结转移, 实性成分三维指标, Mixed ground-glass nodules, Lymph node metastasis, Ratio of solid component

## Abstract

**背景与目的:**

部分胸部计算机断层扫描（computed tomography, CT）上表现为混合磨玻璃结节（mixed ground-glass nodules, mGGNs）的浸润性腺癌（invasive adenocarcinoma, IAC）会出现淋巴结转移，需由亚肺叶切除及纵隔淋巴结采样的术式改为肺叶切除及纵隔淋巴结清扫，故术前进行淋巴结转移的评估对指导手术切除范围及患者预后非常重要。本研究在病理为IAC的大样本mGGN队列中，探索能够预测淋巴结转移的临床和影像学指标，构建mGGN合并淋巴结转移的评估模型。

**方法:**

通过收集北京大学人民医院胸外科2014年1月-2019年10月收治的患者信息，筛选胸部CT病变表现为mGGN且术后病理证实为IAC的患者。统计入组患者的临床信息、影像学信息和淋巴结转移状态，并使用基于人工智能技术的肺结节辅助诊断系统（InferRead CT Lung）获取病例的平均密度、实性成分体积、实性成分百分比、质量等三维度量指标，构建CT密度直方图信息。通过应用R软件建立Lasso逻辑斯蒂回归模型分析评估临床影像学指标与淋巴结转移的相关性。

**结果:**

研究共纳入883例mGGN患者，其中12例（1.36%）出现淋巴结转移。mGGN中的临床影像信息与淋巴结转移的Lasso回归模型分析显示，既往恶性肿瘤病史、平均密度、实性成分平均密度、毛刺征和三维实性成分百分比具有参考意义。基于Lasso回归模型结果建立mGGN中淋巴结转移的评估模型，曲线下面积为0.899。

**结论:**

临床信息结合CT影像信息可以较准确评估mGGN的淋巴结转移情况。

## Abstract (en)

**Background and objective:**

Previous studies have shown that lymph node metastasis only occurs in some mixed ground-glass nodules (mGGNs) which the pathological results were invasive adenocarcinoma (IAC). However, the presence of lymph node metastasis leads to the upgrading of tumor-node-metastasis (TNM) stage and worse prognosis of the patients, so it is important to perform the necessary evaluation before surgery to guide the operation method of lymph node. The aim of this study was to find suitable clinical and radiological indicators to distinguish whether mGGNs with pathology as IAC is accompanied by lymph node metastasis, and to construct a prediction model for lymph node metastasis.

**Methods:**

From January 2014 to October 2019, the patients with resected IAC appearing as mGGNs in computed tomography (CT) scan were reviewed. All the lesions were divided into two groups (with lymph node metastasis or not) according to their lymph node status. Lasso regression model analysis by applying R software was used to evaluate the relationship between clinical and radiological parameters and lymph node metastasis of mGGNs.

**Results:**

A total of 883 mGGNs patients were enroled in this study, among which, 12 (1.36%) showed lymph node metastasis. Lasso regression model analysis of clinical imaging information in mGGNs with lymph node metastasis showed that previous history of malignancy, mean density, mean density of solid components, burr sign and percentage of solid components were informative. Prediction model for lymph node metastasis in mGGNs was developed based on the results of Lasso regression model with area under curve=0.899.

**Conclusion:**

Clinical information combined with CT imaging information can predict lymph node metastasis in mGGNs.

胸部计算机断层扫描（computed tomography, CT）表现为磨玻璃结节（ground-glass nodule, GGN）的肺癌预后相对较好，存在淋巴结转移的比例低，常采取亚肺叶切除及纵隔淋巴结采样。病理为浸润性腺癌（invasive adenocarcinoma, IAC）的部分混合磨玻璃结节（mixed ground-glass nodules, mGGNs）患者会出现淋巴结转移。淋巴结转移会导致患者肿瘤原发灶-淋巴结-转移（tumor-node-metastasis, TNM）分期的升级及预后变差，需接受肺叶切除及纵隔淋巴结清扫。研究^[1⇓⇓-4]^表明术中淋巴结评估与术后漏气时间延长、房性心律不齐、乳糜胸、喉返神经损伤、术中出血增加、手术时间延长、中转开胸的概率增加、住院时间延长等情况有关。考虑到淋巴结病理分期的重要性和淋巴结评估手术方式带来的手术并发症风险，术前进行淋巴结的准确评估以指导手术切除范围和患者预后非常重要。作为放射组学的一种方便而有代表性的方法，CT直方图可以提供一些超出人眼的关键放射学因素，如偏度和熵等^[5⇓-7]^ 。最近，基于体素的胸部CT直方图分析在识别病理侵袭性、淋巴结状态预测和适合亚肺切除的早期肺腺癌方面显示出巨大的作用^[8⇓-10]^。既往对GGN淋巴结转移的预测研究较少，本研究拟在病理为IAC的大样本mGGN队列中，利用基于人工智能的肺结节辅助诊断系统分析mGGN的全面影像学指标，探索能够预测淋巴结转移的临床和影像学指标，构建mGGN淋巴结转移状态的预测模型。

## 1 资料与方法

### 1.1 患者信息及纳入标准

本研究纳入了2014年1月-2019年10月于北京大学人民医院胸外科行手术治疗的患者。纳入标准：（1）胸部CT提示mGGN（直径5 mm-30 mm）并接受手术；（2）术后病理结果证实为IAC；（3）未合并恶性实性结节的肺结节883例。其中570例（64.55%）与313例（35.45%）患者分别接受系统性淋巴结采样或清扫。通过电子病历系统收集对应的临床信息、病理组织学信息及影像学信息，本研究已通过北京大学人民医院伦理委员会批准。

### 1.2 影像学评估及指标的测量和计算

所有患者的胸部CT扫描图像层厚为1 mm或1.25 mm，其中肺窗的窗位-600 HU、窗宽1,500 HU，纵隔窗的窗位40 HU、窗宽400 HU。将所有薄层图像传至肺结节人工智能辅助诊断系统（InferRead CT Lung，推想医疗科技股份有限公司），完成所有肺结节的自动检测。由两位有经验的影像科医生对推想系统筛选出的结节进行单独核查，人工筛选出手术所切除的mGGN。

肺结节人工智能辅助诊断系统基于深度学习算法实现磨玻璃结节范围的自动分割和典型征象的自动识别。首先，以-300 HU为区分实性成分与磨玻璃成分的CT值阈值，基于体素法计算结节体积、平均密度、实性成分体积、实性成分比值（consolidation tumor ratio, CTR）、质量等三维度量指标。接着，根据结节范围内每个CT值所对应的体素数量，构建CT密度直方图，通过Python编码、按照相应的公式自动计算得到包括方差、偏度、峰度、熵等密度直方图相关指标。同时，将该系统检测识别的典型征象经影像科医生确认后作为形态学指标，包括分叶征、毛刺征和胸膜牵拉征等。

### 1.3 统计学分析

使用SPSS 26.0软件进行统计学分析，分类变量采用Fisher精确检验，连续变量中符合正态分布的采用独立样本t检验分析，采用均数±标准差（Mean±SD）表示。不符合正态分布的数据采用秩和检验（曼-惠特尼U检验）分析，采用中位数（四分位间距）[Median (IQR)]表示。应用R软件建立Lasso逻辑斯蒂回归模型，惩罚系数λ取lambda.min，筛选和确定保留的预测因素，进行受试者工作特征（receiver operating characteristic, ROC）曲线验证模型的准确性，并计算ROC曲线下面积以评估预测模型的效用。

## 2 结果

### 2.1 mGGN中不同淋巴结状态的临床病理信息

本研究中术后病理结果为IAC的单纯mGGN患者共883例，其中12例（1.4%）出现淋巴结转移。在病理为IAC的mGGN中，女性占多数（65.0%），只有相对少数的部分合并有吸烟史（20.8%）、既往恶性肿瘤病史（7.2%）和恶性肿瘤家族史（13.4%）。根据mGGN病理淋巴结转移的状态，本研究将其分为无淋巴结转移即LN（-）组和伴有淋巴结转移即LN（+）组，两组的临床信息见表1。两组对比发现，LN（+）组相较于LN（-）组患者存在既往其他恶性肿瘤病史的概率更高（25.0% vs 7.0%, P=0.050），但无统计学意义。两组间的男性比例（33.3% vs 35.0%, P>0.999）、年龄[（64.75±11.76）岁 vs （60.21±9.91）岁，P=0.116]、吸烟史比例（16.7% vs 20.9%, P>0.999）和恶性肿瘤家族史比例（16.7% vs 13.3%, P=0.668）的差异没有统计学意义。

**表 1:** 883例mGGN中不同淋巴结转移状态的临床信息对比

Features	LN (-) (n=871)	LN (+) (n=12)	P
Gender			>0.999
Female	566 (65.0%)	8 (66.7%)	
Male	305 (35.0%)	4 (33.3%)	
Age (yr, Mean±SD)	60.21±9.91	64.75±11.76	0.116
Smoking history			>0.999
Non-smokers	689 (79.1%)	10 (83.3%)	
Current/previous smokers	182 (20.9%)	2 (16.7%)	
Past history of malignancy			0.050
Yes	61 (7.0%)	3 (25.0%)	
No	810 (93.0%)	9 (75.0%)	
Family history of malignant tumors			0.668
Yes	116 (13.3%)	2 (16.7%)	
No	755 (86.7%)	10 (83.3%)	

### 2.2 mGGN中不同淋巴结状态的影像学信息

在评估mGGN的影像学指标中，我们纳入了密度、体积、质量、CTR等三维度量指标，分叶征、毛刺征、胸膜牵拉征等形态学指标以及方差、峰度、偏度和熵等CT直方图相关指标。因既往研究显示mGGN中实性成分显著影响其病理状态，我们将实性成分的三维度量指标单独分析，两组的影像学信息详见表2。

**表 2:** 883例mGGN中不同淋巴结转移状态的影像学信息对比

Features	LN (-)	LN (+)	P
Average density (HU)	-422.82 (181.42)	-185.07 (92.58)	<0.001
Solid mean density (HU)	-172.66 (75.30)	-101.31 (57.28)	<0.001
Volume (cm^3^)	1.49 (1.91)	1.81 (1.67)	0.584
Volume of solid components (cm^3^)	0.27 (0.67)	1.04 (1.58)	0.001
Mass (g)	0.84 (1.19)	1.43 (1.67)	0.101
Mass of solid components (g)	0.22 (0.59)	0.93 (1.47)	0.001
lobar sign	671 (77.0%)	12 (100.0%)	0.079
Spiculation sign	631 (72.4%)	12 (100.0%)	0.044
Pleural traction sign	208 (23.9%)	6 (50.0%)	0.046
Percentage of solid components (%)	21.93 (31.94)	71.90 (21.04)	<0.001
Diameter (mm)	18.12 (8.78)	20.77 (5.56)	0.081
Density histogram			
Variance (×10,000)	2.23 (1.40)	2.52 (1.72)	0.070
Skewness	0.70±0.53	-0.001±0.53	<0.001
Kurtosis	2.72 (1.51)	1.98 (0.62)	0.006
Entropy	8.69 (0.59)	8.80 (0.57)	0.109

Data are expressed as Mean±SD or Median (IQR) or a number (percentage).

LN（+）组相较于LN（-）组的平均密度[-185.07 (92.58) HU vs -422.82 (181.42) HU, P<0.001]、实性成分平均密度[-101.31 (57.28) HU vs -172.66 (75.30) HU, P<0.001]、实性成分体积[1.04 (1.58) cm^3^ vs 0.27 (0.67) cm^3^, P=0.001]、实性成分质量[0.93 (1.47) g vs 0.22 (0.59) g,P=0.001]、实性成分百分比[71.90% (21.04%) vs 21.93% (31.94%), P<0.001]等度量指标均相对较大。在形态学指标中，LN（+）组相较于LN（-）组的毛刺征（100.0% vs 72.4%, P=0.044）和胸膜牵拉征（50.0% vs 23.9%, P=0.046）出现的频率较高。在CT直方图的相关变量中，LN（+）组比LN（-）组的偏度（-0.001±0.53 vs 0.70±0.53, P<0.001）和峰度[1.98 (0.62) vs 2.72 (1.51),P=0.006]的值相对更小。但是两组间的体积[1.81 (1.67) cm^3^ vs 1.49 (1.91) cm^3^, P=0.584）、质量[1.43 (1.67) g vs 0.84 (1.19) g,P=0.101]、分叶征（100.0% vs 77.0%, P=0.079）、直径[20.77 (5.56) mm vs 18.12 (8.78) mm,P=0.081]、方差（×10,000）[2.52 (1.72) vs 2.23 (1.40),P=0.070]和熵[8.80 (0.57) vs 8.69 (0.59),P=0.109]的差异未呈现出统计学意义。

### 2.3 mGGN的临床影像信息与淋巴结转移的Lasso逻辑斯蒂回归模型分析

采用Lasso回归模型对mGGN的临床影像信息所有相关的变量进行筛选，结果显示，随着λ值增大，模型压缩程度越大，进入模型的自变量个数减少，当λ取最小值0.0045时，在所有特征变量的基础上缩减为5个潜在的预测因素，包括既往恶性肿瘤病史、平均密度、实性平均密度、毛刺征、实性成分百分比，并且在Lasso回归模型中具有非零系数（图1，表3）。

**图1:**
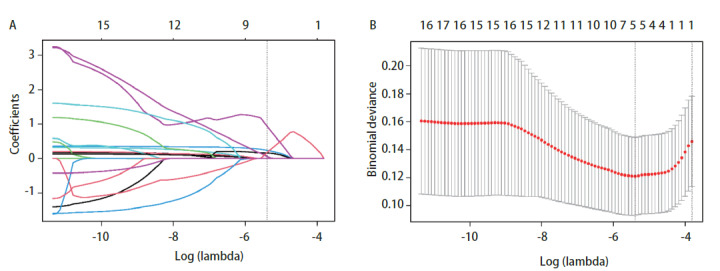
mGGN淋巴结转移的Lasso回归模型分析。 A ：每一条曲线展示了每一个自变量回归系数的变化轨迹；B ：展示了在Lasso回归模型中通过交叉验证和最小准则来确定最优λ值的过程。

**表3:** mGGN中的临床影像信息与淋巴结转移的Lasso回归模型分析

Feature	Coef
Intercept	-5.08865293
Past history of malignancy	0.23230095
Average_density	0.93838018
Solid mean density	0.15383001
Spiculation sign	0.06018955
Percentage of solid components	0.16543674

通过ROC曲线分析Lasso逻辑斯蒂回归模型的预测效能，结果显示曲线下面积为0.899（95%CI: 0.807-0.992），敏感性为87.37%，特异性为83.33%（图2）。

**图2:**
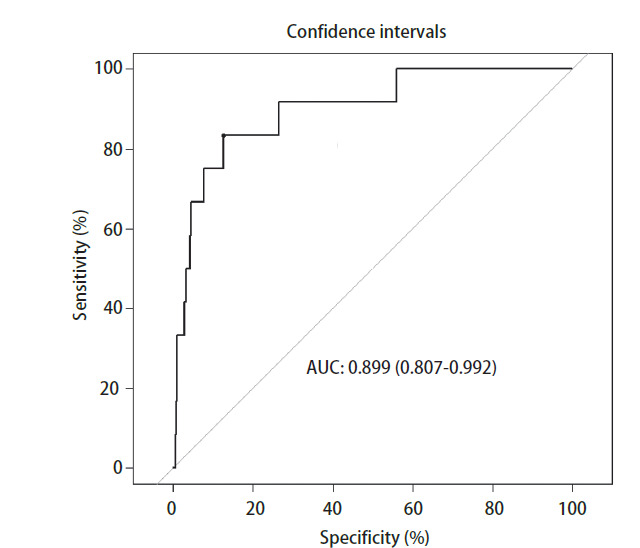
mGGN淋巴结转移的评估模型的ROC曲线

## 3 讨论

为了选择更合适的淋巴结评估方式，术前通过临床影像信息对淋巴结转移进行预测是可行的方法。已经有研究^[11]^证明，pGGN中不会出现淋巴结转移，mGGN中淋巴结转移率（N1/N2）为2.2%。在我们的研究中，整体的淋巴结转移率为2.0%（18/889），但是经排除合并实性结节后，最终淋巴结转移率为1.4%（12/883），与既往报道较为相符。从病理类型来说，原位腺癌（adenocarcinoma in situ, AIS）和微浸润性腺癌（minimally invasive adenocarcinoma, MIA）均未报道出现淋巴结转移，为避免偏倚，本研究在分析中只纳入最终病理证实为IAC的mGGN。

Koike等^[12]^报道年龄与病理淋巴结转移的发生率相关。Pani等^[13]^提出，既往恶性肿瘤病史和身体质量指数（body mass index, BMI）与淋巴结转移独立相关。然而这两项研究的对象为早期肺癌，包括实性结节和GGN。在mGGN的研究中，Cho等^[14]^虽然统计了年龄、性别、吸烟史、既往肺部手术病史或肺癌病史等临床信息，但是这些指标与淋巴结转移均无显著相关性。我们的研究结果显示，既往恶性肿瘤病史是淋巴结转移的预测因素。其原因存在局限性，可能与研究对象的结节类型和病理类型有关，仍然需要大样本量验证。

随着影像学技术的发展和计算水平的提高，三维度量指标逐渐成为了重要的肺结节评估指标，借助计算软件辅助测量肺结节三维度量指标成为新的研究问题^[15]^。既往研究^[12,16,17]^中，肺结节的实性成分大小或肿瘤CTR被认为与淋巴结转移相关，但其研究均为一维度量指标。我们的研究是通过基于人工智能技术计算对mGGN的三维度量指标进行了研究，发现平均密度、实性成分平均密度、实性成分体积、实性成分质量以及三维CTR均与mGGN淋巴结转移呈正相关，其中实性平均密度、三维CTR在Lasso回归分析中对淋巴结转移有预测意义。在我们的研究中，12例淋巴结转移的mGGN中最低三维CTR为16.8%。在一维评估指标相关的研究中，CTR≤0.5的肿瘤均未出现淋巴结转移^[110]^，如果我们假设mGGN的整体和实性成分均为完美的球体，CTR的0.5对应三维CTR的12.5%，低于上述最低的三维CTR为16.8%，与之前的研究结果相符。对于三维实性成分小于该数值的mGGN，可以考虑采用淋巴结采样等创伤性较小的方式以降低患者的手术风险。

既往肺癌的淋巴结转移与影像学检查中的形态学指标的相关性研究相对较少。2014年的一项研究^[18]^表明，在IA期肺腺癌中，支气管充气征的存在与淋巴结转移阴性相关，但在多因素分析中没有意义，同年Hattori等^[19]^报道支气管充气征在纯实性结节中是淋巴结转移阴性的独立预测因素，提示该指标更可能在实性结节中与淋巴结转移相关。mGGN与淋巴结转移的研究本身较少，本研究纳入了常见三种形态学指标，其中毛刺征和胸膜牵拉征在LN（+）组出现的频率较高，毛刺征在Lasso回归分析中对淋巴结转移有预测意义。Shimada等^[20]^在对临床IA期非小细胞肺癌（non-small cell lung cancer, NSCLC）的淋巴结转移的研究中，将肿瘤的CT直方图信息（包括偏度、峰度）纳入进来，这项研究中，偏度在单因素分析中与淋巴结转移相关，但是在多因素分析中无统计学意义。本研究得到了与前述研究相同的结论，但目前在分析中未能观察到CT直方图指标在评估淋巴结转移中的相关性。尽管既往研究^[21]^认为在NSCLC中，偏度提示了脉管和脏层胸膜侵犯，但是从目前的结果来看，CT直方图相关指标无法单纯评估mGGN中淋巴结转移的状态。

本研究证实，既往恶性肿瘤病史、平均密度、实性成分平均密度、毛刺征和三维CTR是mGGN患者中预测淋巴结转移的参考因素。临床信息结合CT影像信息可以较准确地评估mGGN的淋巴结转移情况，有助于指导临床治疗决策尤其是确定手术切除范围。
